# Nitrite-producing oral microbiome in adults and children

**DOI:** 10.1038/s41598-020-73479-1

**Published:** 2020-10-06

**Authors:** Yuria Sato-Suzuki, Jumpei Washio, Dimas Prasetianto Wicaksono, Takuichi Sato, Satoshi Fukumoto, Nobuhiro Takahashi

**Affiliations:** 1 Division of Oral Ecology and Biochemistry, Tohoku University Graduate School of Dentistry, 4-1, Seiryo-machi, Aoba-ku, Sendai, Miyagi Japan; 2 Division of Pediatric Dentistry, Tohoku University Graduate School of Dentistry, Sendai, Japan; 3 Department of Pediatric Dentistry, Faculty of Dental Medicine, Universitas Airlangga, Surabaya, Indonesia; 4 Division of Clinical Chemistry, Department of Medical Technology, Niigata University Graduate School of Health Sciences, Niigata, Japan

**Keywords:** Microbiology, Bacteria, Biofilms, Microbial communities, Health care, Dentistry

## Abstract

Recently, it was suggested that the nitrite (NO_2_^−^) produced from NO_3_^−^ by oral bacteria might contribute to oral and general health. Therefore, we aimed to clarify the detailed information about the bacterial NO_2_-production in the oral biofilm. Dental plaque and tongue-coating samples were collected, then the NO_2_-producing activity was measured. Furthermore, the composition of the NO_2_^−^-producing bacterial population were identified using the Griess reagent-containing agar overlay method and molecular biological method. NO_2_^−^-producing activity per mg wet weight varied among individuals but was higher in dental plaque. Additionally, anaerobic bacteria exhibited higher numbers of NO_2_^−^-producing bacteria, except in the adults’ dental plaque. The proportion of NO_2_^−^-producing bacteria also varied among individuals, but a positive correlation was found between NO_2_^−^-producing activity and the number of NO_2_^−^-producing bacteria, especially in dental plaque. Overall, the major NO_2_^−^-producing bacteria were identified as *Actinomyces, Schaalia, Veillonella* and *Neisseria*. Furthermore, *Rothia* was specifically detected in the tongue coatings of children. These results suggest that dental plaque has higher NO_2_^−^-producing activity and that this activity depends not on the presence of specific bacteria or the bacterial compositions, but on the number of NO_2_^−^-producing bacteria, although interindividual differences were detected.

## Introduction

Nitrate (NO_3_^−^) is abundant in green/yellow vegetables (several tens to thousands ppm), especially in leafy vegetables^1,2^. Diet-derived NO_3_^−^ is partly converted by oral bacteria to NO_2_^−^. After being absorbed from the gastrointestinal tract and into the blood, it is gradually oxidized to NO_3_^−^, some of which is secreted into the oral cavity in saliva. This is called “the entero-salivary nitrogen oxide cycle”^3^, and oral bacteria play a major role in the production of NO_2_^−^ from NO_3_^−^ via this cycle. Saliva that is collected directly from the parotid gland contains no NO_2_^−^^4^ and the use of antibacterial antiseptic mouthwash reduces the blood NO_2_^−^ concentration^5^. These observations indicate that oral bacteria are involved in the production of NO_3_^−^ from NO_2_^−^ and that oral bacteria are essential for nitrogen oxide production and circulation. In recent years, NO_2_^−^ has been reported to have antimicrobial effects, e.g., it suppresses the metabolism and growth of caries- and periodontal disease-related bacteria^6–9^, and it is also known to ameliorate cardiovascular disease by improving the systemic blood circulation^10^. These findings suggest that NO_2_^−^ is relevant to not only oral health, but also general health.

In a previous study, the proportion of NO_2_^−^-producing bacteria in the oral biofilm was examined using the metagenomics method^11^. The metagenomic and metatranscriptomic approaches are comprehensive, while their analytical power depends on comprehensiveness of gene database about bacterial species and nitrate-reducing enzymes. In addition, relatedness of metagenomic data and metatranscriptomic data is quite complicated and it is often difficult to get an answer which bacterium is responsible for the targeted phenotype. The culture method has limitations such as growth selection, while this method has also advantage such as isolating bacteria as live cells and confirming their ability of nitrite production from nitrate. In this study, we would like to focus on the bacterial species that works as nitrate reducer in actual in the oral cavity. From these circumstances, we first tried to isolate the nitrite-producing phenotypic bacteria.

In a report using culture-based method, oral biofilm samples that were collected from the tooth surfaces, tongues, cheeks, and palatal mucosae of adults exhibited NO_2_^−^-producing activity, especially those from the tongue surface^12^. *Veillonella*, *Actinomyces*, *Rothia*, and *Staphylococcus* have been identified as the predominant NO_2_^−^-producing bacteria in the oral cavity^12^. In addition, these genera were also detected in the saliva of adults and newborns^13^, and the frequency of *Veillonella* was associated with NO_2_^−^-producing activity in saliva^14^. However, no previous study has examined the NO_2_^−^-producing activity of the oral biofilm per unit wet weight, or interindividual differences in the composition of NO_2_^−^-producing bacterial populations at the bacterial species level. Furthermore, the previous findings that the salivary NO_2_^−^ concentration is higher in children^1^ and that the composition of the oral bacterial population differs between children and adults^11,15^ suggest that the types and proportions of NO_2_^−^-producing bacteria in children are different from those in adults.

Therefore, the purpose of this study was to measure the NO_2_^−^-producing activity of the oral biofilms on the tooth and tongue surfaces of adults and children, and to analyze the bacterial compositions of these biofilms by isolating and identifying NO_2_^−^-producing bacteria using culture-based method for both adults and children.

## Results

### NO_2_^−^-producing activity detected in dental plaque and tongue coatings

**Figure 1:**
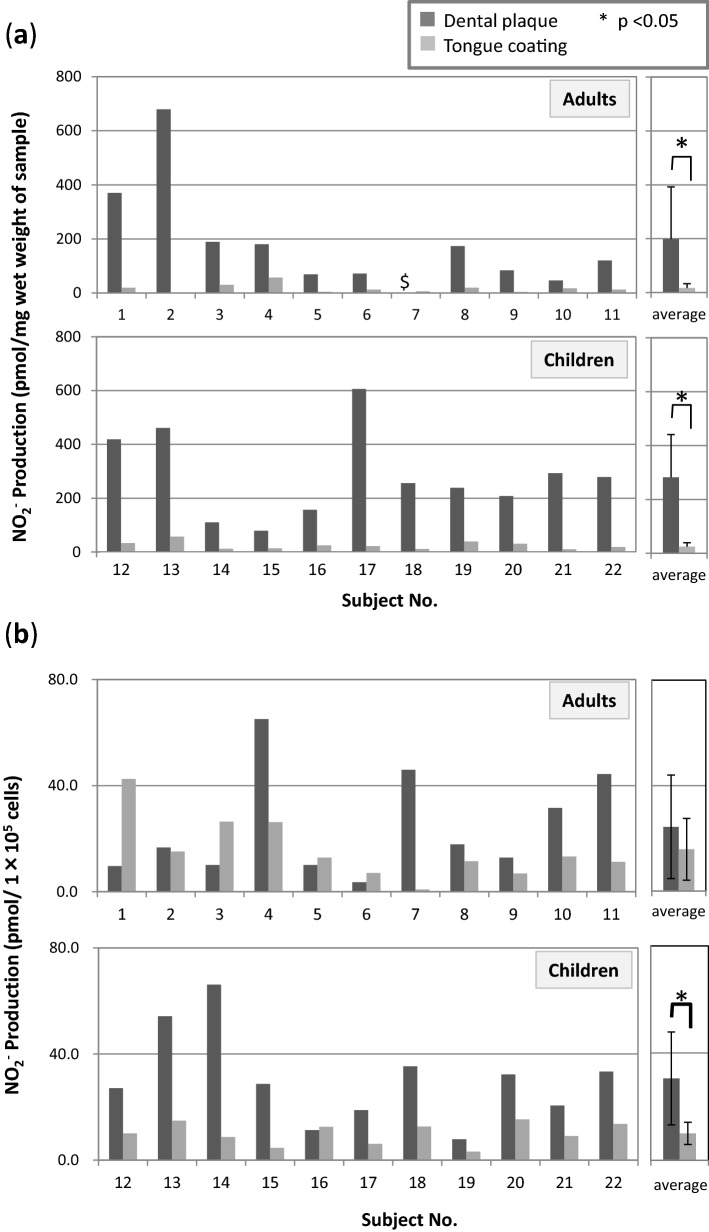
(**a**) The NO_2_^−^-producing activity of dental plaque and tongue-coating samples (pmol/mg wet weight). (**b**) The NO_2_^−^-producing activity of dental plaque and tongue-coating samples (pmol/1 × 10^5^ cells). Subject numbers: 1–11 (adults), 12–22 (children); *significant difference (p < 0.05). $: not available because the wet weight of the sample was below the detectable limit.

The NO_2_^−^-producing activity (per mg wet weight) of each sample was determined (Fig. 1a). There was no significant difference in the mean NO_2_^−^ production value between adults and children, although large interindividual differences were seen. The NO_2_^−^-producing activity of dental plaque was significantly higher than that of tongue coatings in both adults (p = 0.017) and children (p < 0.001). On the other hand, the NO_2_^−^-producing activity per unit cell numbers of each sample was determined (Fig. 1b). Similarly, there was no significant difference in the mean NO_2_^−^ production value between adults and children, although large interindividual differences were seen. However, the NO_2_^−^-producing activity of dental plaque was significantly higher than that of tongue coatings in only children (p = 0.002).

### The number and percentage of NO_2_^−^-producing bacteria in dental plaque and tongue coatings

**Figure 2:**
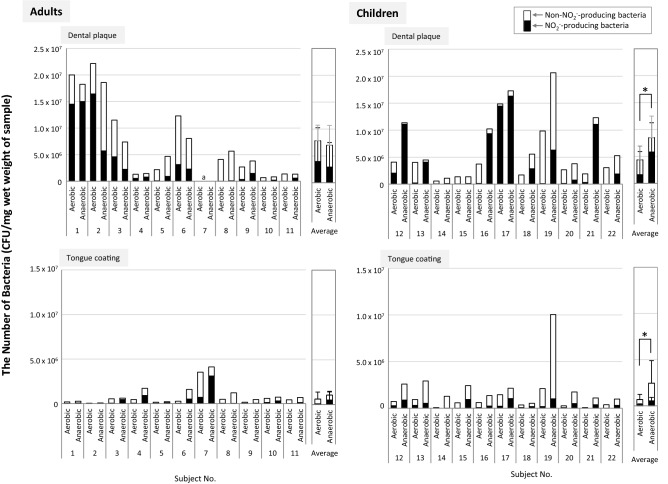
The numbers of NO_2_^−^-producing and non-NO_2_^−^-producing bacteria detected in dental plaque and tongue-coating samples cultured under aerobic and anaerobic conditions (CFU/mg wet weight of sample). Subject numbers: 1–11 (adults), 12–22 (children); Aerobic: the bacteria on the blood agar plate were cultured aerobically; Anaerobic: the bacteria on the blood agar plate were cultured anaerobically; *significant difference (p < 0.05); a: not available because the wet weight of the sample was below the detectable limit.

**Figure 3:**
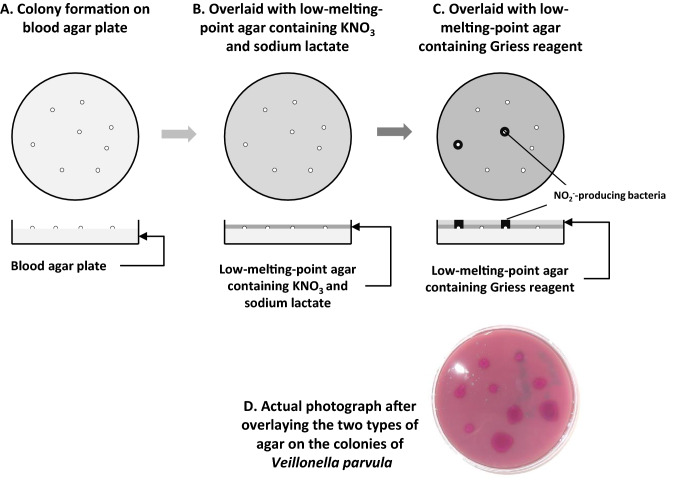
The Griess reagent-containing agar overlay method for detecting NO_2_^−^-producing bacteria.

Dental plaque and tongue-coating samples were cultured on agar plates under aerobic and anaerobic conditions, and the number and percentage of NO_2_^−^-producing bacteria and non-NO_2_^−^-producing bacteria were determined (Fig. 2) using the agar overlay culture method (Fig. 3). The number of NO_2_^−^-producing bacteria (per mg wet weight) was higher in the dental plaque samples than in the tongue-coating samples in both adults and children and under both aerobic and anaerobic conditions. The percentage of NO_2_^−^-producing bacteria was higher in the anaerobic cultures than in the aerobic cultures, except in the case of the adult dental plaque samples (Fig. 2). However, large interindividual differences were again noted, and there were no correlations between the percentage of NO_2_^−^-producing bacteria and oral clinical examination parameters.

### Correlations between the NO_2_^−^-producing activity and the number of NO_2_^−^-producing bacteria in dental plaque and tongue coatings

**Figure 4:**
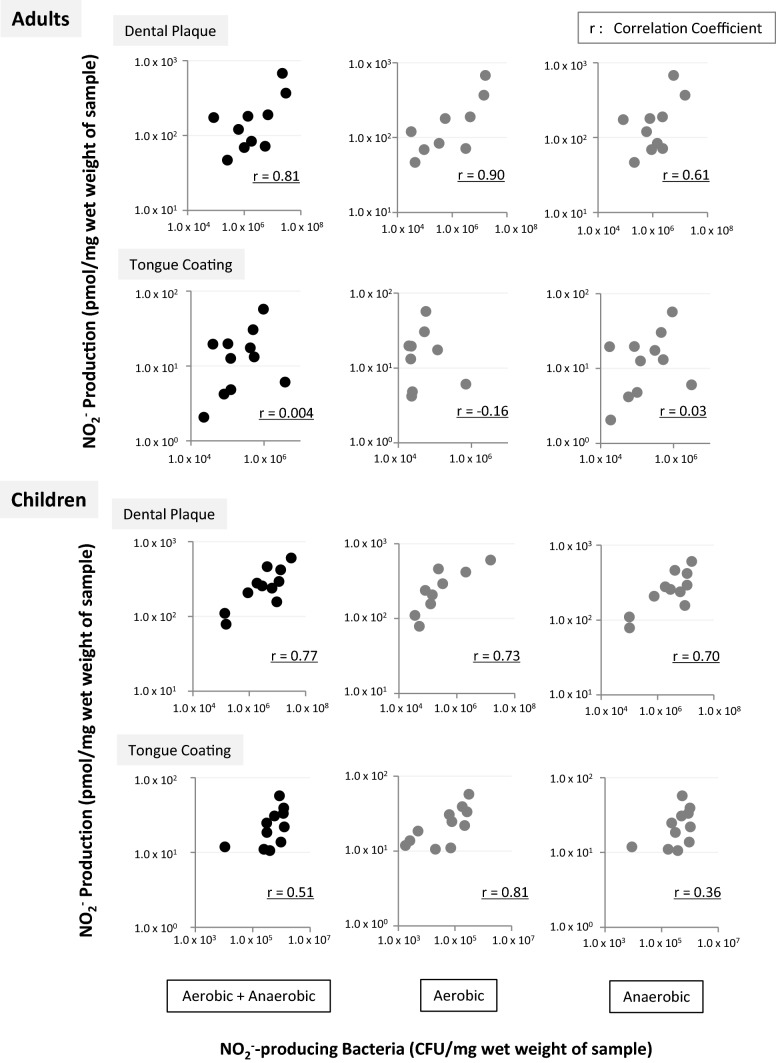
Correlation between NO_2_^−^-producing activity and the number of NO_2_^−^-producing bacteria (aerobic, anaerobic, and aerobic plus anaerobic conditions) in the dental plaque and tongue coating samples from adults and children. Both axes are displayed on logarithmic scales. *r* correlation coefficient, *Aerobic* the bacteria on the blood agar plate were cultured aerobically, *Anaerobic* the bacteria on the blood agar plate were cultured anaerobically.

The correlation between NO_2_^−^-producing activity and the number of NO_2_^−^-producing bacteria in dental plaque (the number of aerobic cultures, the number of anaerobic cultures, and the total number of cultures) was found to be significant in both adults and children (r = 0.61–0.90, p < 0.05) (Fig. 4). In addition, a significant correlation was detected between these parameters for children’s tongue coatings (r = 0.81, p < 0.05), but not adult’s tongue coatings (Fig. 4).

### Identification of NO_2_^−^-producing bacteria

**Figure 5:**
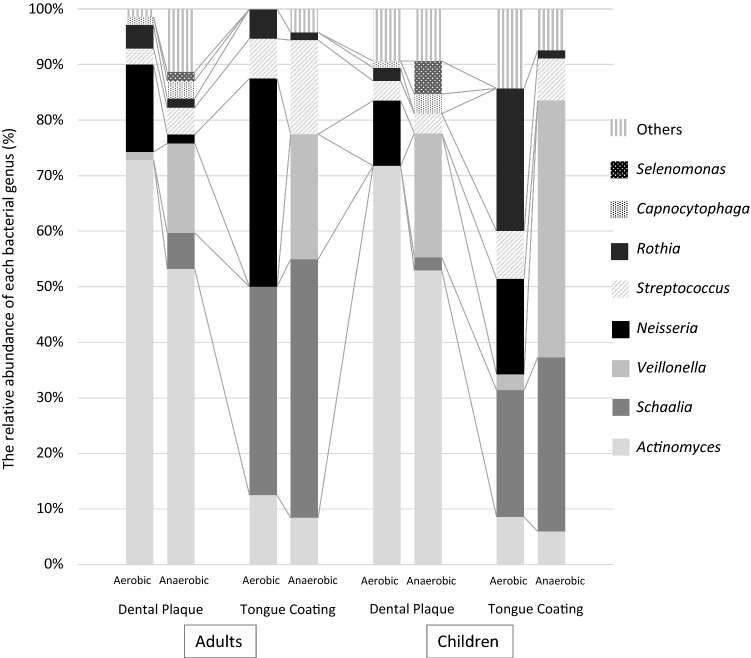
The proportion of NO_2_^−^ -producing bacterial genera identified in the dental plaque and tongue coating samples from adults and children. All data were adopted from Table 1.

Figure 5 shows the proportion of NO_2_^−^-producing bacteria identified. In both adults and children, *Actinomyces, Schaalia* and *Neisseria* accounted for a large proportion of NO_2_^−^-producing bacteria under aerobic conditions, while *Actinomyces, Schaalia* and *Veillonella* accounted for a large proportion of NO_2_^−^-producing bacteria under anaerobic conditions. The genus *Actinomyces* was mainly detected in dental plaque than in tongue coating, and the most common species detected were *Actinomyces naeslundii*, *A. oris*, and *A. johnsonii* (Table 1). On the other hand, the genus *Schaalia* was detected higher in tongue coating. The genus *Neisseria* was mainly found under aerobic conditions, and there was no difference in the frequencies of *Neisseria* species between the dental plaque and tongue coating samples. The genus *Veillonella* was only found under anaerobic conditions, and the most common species in the dental plaque samples was *V. parvula*, and the most common species in the tongue coating samples was *V. dispar*, followed by *V. atypica* and *V. parvula* (Table 1).

**Table 1:** The number and proportion of NO_2_^−^-producing bacteria identified in the dental plaque and tongue coating samples from adults and children.

	Adults				Children			
	Dental plague		Tongue coating		Dental plague		Tongue coating	
	Aerobic	Anaerobic	Aerobic	Anaerobic	Aerobic	Anaerobic	Aerobic	Anaerobic
*Actinomyces naeslundii*	30	18	3	2	24	12	0	0
*Actinomyces oris*	5	11	2	0	28	22	0	0
*Actinomyces johnsonii*	10	2	0	0	8	9	0	0
*Actinomyces graevenitzii*	0	0	2	2	0	0	3	3
*Actinomyces hongkongensis*	1	1	0	0	0	0	0	0
*Actinomyces georgiae*	0	0	0	0	0	2	0	0
*Actinomyces massiliensis*	0	0	0	0	1	1	0	0
*Actinomyces lingnae*	0	0	0	0	0	0	0	1
*Actinomyces oris or naeslundii*	5	1	0	1	0	0	0	0
**Total Actinomyces**	51 (72.9)*	**33 (53.2)**	**7 (12.3)**	**6 (8.2)**	**61 (71.8)**	**45 (52.9)**	**3 (8.6)**	**4 (6.0)**
*Schaalia odontolytica*	0	4	21	33	0	2	8	21
**Total Schaalia**	**0 (0)**	**4 (6.5)**	**21 (36.8)**	**33 (45.2)**	**0 (0)**	**2 (2.4)**	**8 (22.9)**	**21 (31.3)**
*Veillonella parvula*	0	8	0	3	0	19	0	3
*Veillonella dispar*	1	0	0	6	0	0	0	14
*Veillonella atypica*	0	0	0	6	0	0	0	8
*Veillonella tobetsuensis*	0	1	0	0	0	0	1	3
Other Veillonella species	0	1	0	1	0	0	0	3
**Total Veillonella**	**1 (1.4)**	**10 (13.9)**	**0 (0)**	**16 (21.9)**	**0 (0)**	**19 (22.4)**	**1 (2.9)**	**31 (44.9)**
*Neisseria flavescens*	0	0	11	0	0	0	0	0
*Neisseria macacae*	1	0	0	0	8	0	2	0
*Neisseria mucosa*	1	1	2	0	1	0	2	0
*Neisseria oralis*	4	0	0	0	0	0	0	0
*Neisseria subflava*	0	0	3	0	0	0	0	0
*Neisseria elongata*	2	0	0	0	0	0	0	0
*Neisseria sicca*	0	0	0	0	1	0	0	0
Other Neisseria species	3	0	5	0	0	0	2	0
**Total Neisseria**	**11 (15.7)**	**1 (1.6)**	**21 (36.8)**	**0 (0)**	**10 (11.8)**	**0 (0)**	**6 (17.1)**	**0 (0)**
*Streptococcus salivarius*	0	0	1	0	0	0	2	4
*Streptococcus sanguinis*	1	1	0	2	0	1	0	0
*Streptococcus australis*	0	0	0	2	0	2	0	0
*Streptcoccus oralis*	0	0	0	2	1	0	0	0
*Streptcoccus parasanguinis*	0	0	1	1	1	0	0	0
*Streptcoccus mitis*	0	0	0	0	0	0	0	1
*Streptococcus infantis*	0	0	0	1	0	0	0	0
*Streptococcus mutans*	0	0	0	0	1	0	0	0
Other Streptococcus species	1	2	2	4	0	0	1	0
**Total Streptococcus**	**2 (2.9)**	**3 (4.8)**	**4 (7.0)**	**12 (16.4)**	**3 (3.5)**	**3 (3.5)**	**3 (8.6)**	**5 (7.2)**
*Rothia mucilaginosa*	0	0	3	1	0	0	0	1
*Rothia dentocariosa*	2	0	0	0	1	0	1	0
*Rothia aeria*	0	0	0	0	1	0	2	0
Rothia species	1	1	0	0	0	0	0	0
**Total Rothia**	**3 (4.3)**	**1 (1.6)**	**3 (5.3)**	**1 (1.4)**	**2 (2.4)**	**0 (0)**	**9 (25.7)**	**1 (1.4)**
*Capnocytophaga ochracea*	1	0	0	0	0	3	0	0
*Capnocytophaga gingivalis*	0	2	0	0	0	0	0	0
Capnocytophaga genospecies	0	0	0	0	1	0	0	0
**Total Capnocytophaga**	**1 (1.4)**	**2 (3.2)**	**0 (0)**	**0 (0)**	**1 (1.2)**	**3 (3.4)**	**0 (0)**	**0 (0)**
*Selenomonas noxia*	0	1	0	0	0	0	0	0
*Selenomonas flueggei*	0	0	0	0	0	1	0	0
*Selenomonas artemidis*	0	0	0	0	0	4	0	0
**Total Selenomonas**	**0 (0)**	**1 (1.6)**	**0 (0)**	**0 (0)**	**0 (0)**	**5 (5.9)**	**0 (0)**	**0 (0)**
*Paraburkholderia fungorum*	0	0	0	0	3	0	4	1
*Fusobacterium nucleatum*	0	5	0	0	0	2	0	0
*Pseudopropionibacterium propionicum*	0	0	0	0	0	4	0	0
*Haemophilus parainfluenzae*	0	1	0	2	0	0	1	1
*Prevotella melaninogenica*	0	0	0	1	0	0	0	1
*Eikenella corrodens*	0	0	0	0	2	0	0	0
Other species^a^	1	1	0	0	3	2	0	2
**Total others**	**1 (1.4)**	**7 (11.3)**	**0 (0)**	**3 (4.1)**	**8 (9.4)**	**8 (9.4)**	**5 (14.3)**	**5 (7.2)**
**Unknown species**	**0 (0)**	**0 (0)**	**1 (1.8)**	**2 (2.7)**	**0 (0)**	**0 (0)**	**0 (0)**	**0 (0)**
**Total**	**70 (100)**	**62 (100)**	**57 (100)**	**73 (100)**	**85 (100)**	**85 (100)**	**35 (100)**	**67 (100)**

Bold values indicate the total number for each bacterial genus.

*The value in parentheses is percentage in total.

^a^Only once detected bacteria, including *Aggregatibacter segnis, Staphylococcus epidermidis, Moraxella lacunata, Leptotrichia hofstadii, Gemella morbillorum, Campylobacter concisus, Lachnoanaerobaculum umeaense*, and *Morococcus cerebrosus.*

The samples of adults were characterized by a high proportion of *Neisseria* in tongue coatings, whereas the samples of children were characterized by high proportions of *Rothia* in aerobic bacteria and *Veillonella* in anaerobic bacteria detected in tongue coatings. In addition, *Burkholderia* (*Paraburkholderia*), *Selenomonas*, and *Pseudopropionibacterium* were not observed substantially in adults, while they were relatively common in children.

## Discussion

### Modified Griess reagent-containing agar overlay method

Using the Griess reagent-containing agar overlay method, we were able to visually detect colonies of NO_2_^−^-producing bacteria (Fig. 3). The strongest color was seen about 20 min after the application of the Griess reagent-containing agar, and after that the color gradually tended to fade. Therefore, the observations were performed 20 min after the colonies were covered with agar. In addition, normal agar with a melting point of 80–90 °C was replaced by agar with a low melting point of 30–31 °C to prevent heat damage to the bacteria. Furthermore, lactate was added to the covering agar to activate the metabolic activity of lactate-utilizing bacteria, such as *Veillonella*^16,17^ and *Neisseria*^18^. These modifications might have allowed us to isolate new groups of NO_2_^−^-producing bacteria as discussed below.

### The NO_2_^−^-producing activity and number of NO_2_^−^-producing bacteria in adults and children

The present study revealed that NO_2_^−^-producing activity per mg wet weight of sample varied among individuals in both adults and children, but overall, it was suggested that the same wet weight of dental plaque made a greater contribution to NO_2_^−^ production than that of tongue coatings (Fig. 1a). On the other hand, NO_2_^−^-producing activity per 10^5^ bacterial cells in each sample varied among individuals in both adults and children similarly, but the tendency that NO_2_^−^-producing activity of dental plaque was significantly higher than that of tongue coatings was observed in only children, but not in adults (Fig. 1b). The bacterial density in same wet weight of sample was higher in dental plaque than tongue coating except only 1 child subject. Such differences in bacterial cell density may affect the results in this study. However, in some adult subjects, the NO_2_^−^-producing activity per same cell numbers was higher in tongue coating than in dental plaque. The inter-individual differences in the bacterial composition of nitrate reducing bacteria may also affect it, although a further study is needed. The number and percentage of NO_2_^−^-producing bacteria also varied among individuals, but they were higher under anaerobic conditions than under aerobic conditions, except in adult dental plaque (Fig. 2), indicating that facultative anaerobic and obligate anaerobic bacteria are the main bacteria involved in oral NO_2_^−^ production.

Positive correlations between NO_2_^−^-producing activity and the number of NO_2_^−^-producing bacteria were detected for the dental plaque of adults and children, and some of the children’s tongue-coating samples (Fig. 4). These results suggest that the NO_2_^−^-producing activity of dental plaque in adults and children and tongue-coating in children is basically determined by the number of NO_2_^−^-producing bacteria. On the other hand, the significant correlation was not detected for tongue coating of adults. We think that multiple nitrite-producing bacteria, rather than a few limited bacteria, may contribute to nitrate reduction in the oral cavity, but we have limited information about the relative nitrate reducing activity of each bacterial species in the oral cavity. To clarify why the correlation was not detected in some cases in Fig. 4, we need to consider the differences of the bacterial composition and the nitrite producing ability of each bacterial species. Furthermore, in a recent research, the NO_2_^−^ producing activity of *Veillonella* species was much affected by oral environmental factors such as pH, lactate concentration^19^. Thus, we maybe should consider such effects when we evaluate the NO_2_^−^ production in the oral cavity.

In all of the subjects used in this study were in good oral health, and the exclusion criteria included factors such as untreated dental caries and untreated periodontal diseases. Because there was not much differences among subjects in clinical parameters, the correlations between NO_2_^−^-producing activity and oral clinical factors may not be fully analyzed. We would like to clarify this correlation as a future research.

### NO_2_^−^-producing bacterial species in adults and children

*Actinomyces* accounted for the largest proportion of NO_2_^−^-producing bacteria in both aerobic and anaerobic conditions, particularly in dental plaque (Fig. 5). *Actinomyces* has been reported to be the major type of oral NO_2_^−^-producing bacteria^12^, and the present study supports this finding. The next most common bacterial genus was *Schaalia*, especially *Schaalia odontolytica* was detected higher in tongue coatings (Table 1). *Schaalia odontolytica* has been proposed as *Actinomyces odontolyticus*, and re-named in 2018^20^. *Schaalia odontolytica* has been also reported to be the major type of oral NO_2_^−^-producing bacteria as *Actinomyces odontolyticus*^12^. Another most common bacterial genus was *Veillonella*, especially in the tongue coatings of children (Fig. 5), which is consistent with the findings of Doel et al. (2005) and Hyde et al. (2014). Mashima et al. (2017) also reported *Veillonella* as a major bacterial genus in children’s saliva.

The present study supports the previous findings that *Neisseria* was one of the predominant NO_2_^−^-producing bacteria in the oral cavities of adults^9,11^. Furthermore, it was the first study to show that *Neisseria* accounted for a relatively high percentage of NO_2_^−^-producing bacteria in children (Fig. 5). However, *Neisseria* was not detected in a previous study in which the agar overlay method was used to detect NO_2_^−^-producing bacteria^12^. This might have been due to individual differences between the subjects, but it could also have been due to our use of covering agar with a low melting point (30–31 °C). The use of such agar might have helped to prevent the heat damage associated with covering samples with normal agar with a melting point of 80–90 °C, although the heat sensitivity of the NO_2_^−^-producing activity of *Neisseria* has not been confirmed. Moreover, the addition of lactate to the covering agar in the present study might have promoted the NO_2_^−^-producing activity of *Neisseria*, as well as lactate-utilizing bacteria (*Veillonella*), since *Neisseria* was also reported to utilize lactate as an energy source^18^. The modified agar overlay method used in the current study might be more useful for detecting NO_2_^−^-producing bacteria.

*Rothia* is an NO_2_^−^-producing bacterium that is found in the oral cavity^9,12,13^. The present study showed that *Rothia* accounted for a high proportion of the NO_2_^−^-producing bacteria in children’s tongue coatings (Fig. 5). Mashima et al. (2017) also detected *Rothia* in the oral cavities of children, although they did not examine NO_2_^−^-producing activity. The finding that *Rothia* is involved in the NO_2_^−^-producing activity of tongue coatings in children is new, and further research is needed on the activity of this genus.

*Paraburkholderia* was the first NO_2_^−^-producing bacterium detected in children (Fig. 5). Interestingly, Coenye et al. reported that *Burkholderia*, which is closely related to *Paraburkholderia*, displays NO_2_^−^-producing activity^21^. However, *Paraburkholderia* is not reported yet to exist in the oral cavity, but is reported to be detected in plants^22^. Food debris in samples may contain this bacterium in this study.

The other bacterial genus detected in this study, *Streptococcus, Capnocytophaga, Selenomonas, Haemopholus, Prevotella and Eikenella*, were also reported to be positive in nitrate reduction in previous reports^11,12^ or to have the related gene of nitrate reduction. *Propionibacterium acnes* is also reported to have nitrite reductase^23^. Genus *Pseudopropionibacterium* detected in this study belonged formerly to this genus *Propionibacterium*, hence this genus also may have nitrate reductase. *Fusobacterium nucleatum* has been known to have nitrite reductase but not nitrate reductase^24^, therefore we thought the possibility of “false-positive in this method” about the detection of this species.

The culture-based method has a limitation to clarify the comprehensive microbial diversity compared with metagenomics-based method, and also the results may be affected by the differences of culture conditions. The present data basically supported the data in the previous studies^11,12^ at genus and species level, although there is a little difference. These factors on the methods may affect the results, although the difference of subjects may affect it.

### Ecological considerations regarding NO_2_^−^-producing bacteria and their relationships with oral and general health

Our results indicate that *Actinomyces, Schaalia* and *Veillonella* are the main contributor to NO_2_^−^-producing in dental plaque and tongue coatings. In addition, *Neisseria* is more common in adults’ tongue coatings, and *Rothia* is more common in children’s tongue coatings. *Actinomyces* and *Schaalia* are facultative anaerobes, and *Veillonella* is an obligate anaerobe, while *Neisseria* and *Rothia* are aerobes. The detection of all of these bacteria suggests that they are segregated in different parts of the biofilm with different oxygen concentrations, but coexist through metabolic networks. The bacterial production of NO_2_^−^ from NO_3_^−^ is usually catalyzed by reducing power, which is supplied by bacterial metabolism. *Actinomyces* and *Schaalia* can metabolize carbohydrates, which produces lactate and reducing power, while *Veillonella* can metabolize lactate^25^, which produces reducing power and stimulates their metabolism^26^. Lactate can also be produced by most saccharolytic bacteria in the oral cavity, such as streptococci and lactobacilli.

Furthermore, *Actinomyces* is also capable of utilizing lactate under aerobic conditions, which produces reducing power^27^, suggesting that it has the potential to produce NO_2_^−^ via a pathway coupled with the aerobic lactate metabolism. *Neisseria* is also known to utilize lactate via a pathway that produces reducing power^18^. However, there is limited information about the metabolic properties of *Rothia*, and further metabolic studies of this genus and the other oral NO_2_^−^-producing bacteria identified in the present study are needed. It was not possible to explain the differences in the composition of the NO_2_^−^-producing bacterial populations between adults and children or among individuals in the current study, but the metabolic characteristics and networks of the oral microbiome^28^ might account for these variations. Rosier et al. suggested that nitrite leads to rapid modulation of microbiome in their latest report^9^, various environmental factors such as pH, oxygen concentrations in the oral cavity may also affect the bacterial composition of oral microbiome. Although it is difficult to discuss the detailed effects by these environmental factors on the differences of oral microbiome at the present stage, further study is essential.

NO_2_^−^ has attracted attention due to its antibacterial activity and its ability to suppress cardiovascular disease by improving the systemic blood circulation^10^. A double-blind study found that cardiovascular disease symptoms were ameliorated in individuals that consumed NO_3_^−^-rich foods^29^. Therefore, it might be possible to prevent both oral (such as caries and periodontitis) and cardiovascular disease by using NO_2_^−^-producing bacteria as probiotics and/or NO_3_^−^ as a prebiotic^30^. Furthermore, the associations between NO_2_^−^ and methemoglobinemia or the onset of cancer are unclear and require further study^31^.

## Materials and methods

This study was approved by the Research Ethics Committee of Tohoku University Graduate School of Dentistry (approval number: 2018-3-17). All methods were carried out in accordance with relevant guidelines and regulations.

### Subjects

Twenty-two volunteers (11 adults [subject no. 1–11], 22–43 years; 11 children [subject no. 12–22], 5–12 years) were included in this study, and informed consent was obtained from each subject. The exclusion criteria were as follows: no dental plaque or tongue coating being seen during a visual inspection; toothbrushing after meals; eating or drinking anything other than water or sugar-free drinks after meals; having edentulous jaws, systemic disease, untreated dental caries, untreated periodontal disease, or oral mucosal lesions; having used a mouthwash for disinfection within 24 h; and taking antibiotics within 3 months. DMFT, dmft, and OHI-S were recorded as oral clinical examination parameters.

### Sample collection and preparation

Samples were collected from adults at the Division of Oral Ecology and Biochemistry, Tohoku University Graduate School of Dentistry, and they were collected from children at the Department of Pediatric Dentistry, Tohoku University Hospital. The volunteers were asked to stop brushing their teeth in the morning, and dental plaque and tongue coating samples were collected ≥ 2 h after meals. Sampling was performed by a same dentist. Dental plaque samples were collected from the full dentition using a sterile toothpick or a sterile excavator and placed in a sterile tube on ice. Tongue coatings were collected from the tongue dorsum using a sterile wooden spatula, suspended in a sterile tube containing 1 mL of ice-cold physiological saline, and centrifuged at 10,000 rpm and 4 °C for 2 min (H-15FR, Kokusan, Tokyo, Japan). The resultant pellets were used as tongue coating samples. The wet weight of the dental plaque and tongue-coating samples was measured, and then the samples were suspended in 1 mL of 40 mM phosphate buffer solution (PPB) (pH 7), homogenized using a sterile glass homogenizer for 5 min, and used for the subsequent experiments.

### Measurement of NO_2_^−^-producing activity in dental plaque and tongue coating samples

The reaction mixture used to assess NO_2_^−^-producing activity contained 100 μL of the dental plaque or tongue coating suspension, 10 µL of 1 M PPB (pH 7.0), 10 μL of 25 mM KNO_3_, and 130 μL of de-ionized water. After being subjected to aerobic incubation at 37 °C for 30 min, the reaction mixture was centrifuged at 10,000 rpm at 4 °C for 3 min, and the supernatants were used for the NO_2_^−^ assay^32^.

The NO_2_^−^ concentration was measured with the Griess reagent kit (Dojindo Molecular Technologies, Kumamoto, Japan), in which naphthylethylenediamine dihydrochloride and sulphanilamide reacts with NO_2_^−^ to form a purple azo product. The concentration of the azo product was measured colorimetrically at 540 nm using a microplate reader (Varioskan Flash, Thermo Fisher Scientific, Tokyo, Japan).

## Culturing of bacteria

Dental plaque and tongue coating suspensions that had been diluted ten-fold in sterilized 40 mM PPB (pH 7.0) were prepared, spread onto blood agar plates (CDC anaerobe 5% sheep blood agar, BD Japan, Japan), and cultured at 37 °C aerobically in an incubator (CL-410, Advantec Co. Ltd., Tokyo, Japan) or anaerobically in the anaerobic chamber containing 80% N_2_, 10% CO_2_, and 10% H_2_ (ANB-18-2E, Hirasawa, Tokyo, Japan). After the samples had been cultured for 1 week, the total number of colonies on each plate was counted, and the number of bacteria in each sample was calculated. NO_2_^−^-producing bacterial colonies on the same agar plates were detected using the method described below.

### Detection of NO_2_^−^-producing bacteria

NO_2_^−^-producing bacterial colonies were detected by covering the plates with agar containing Griess reagent (Fig. 3)^12^. The blood agar plates were covered with agar containing 1 mM KNO_3_ and 10 mM sodium lactate. After the agar had hardened, the surface was covered with agar containing naphthylethylenediamine dihydrochloride and sulphanilamide, the components of Griess reagent. In the modified method used in the present study, lactate was added to activate the metabolism of lactate-utilizing bacteria, such as *Veillonella*^16,17^ and *Neisseria*^18^, and the covering agar was replaced by agar with a low melting point (30–31 ℃) (Nakalai Tesque, Kyoto, Japan) to avoid heat damage to the bacteria. After being incubated for 20 min at room temperature, NO_2_^−^-producing bacterial colonies created purple-colored spots, which were easy to identify (Fig. 3).

### Identification of NO_2_^−^-producing bacteria

The detected NO_2_^−^-producing bacteria were identified using a molecular biological approach, which was described in a previous report^33^. Genomic DNA was extracted from bacterial colonies using the InstaGene matrix kit (Bio-Rad, California, USA), according to the manufacturer’s instructions. Next, the 16S rRNA gene sequence was amplified using the polymerase chain reaction (PCR) using the universal primers 27F and 1492R^34^ and Taq DNA polymerase (HotStarTaq Master Mix, Qiagen, Netherlands), according to the manufacturer’s instructions. The primer sequences were as follows: 27F, 5′-AGAGTTT GATCMTGGCTCAG-3′; and 1492R, 5′-TACGGYTACCTTGTTAC GACTT-3′. Amplification was performed using a PCR Thermal Cycler MP (TaKaRa Biomedicals, Japan) and the following program: 15 min at 95 °C for the initial heat activation and 35 cycles of 1 min at 94 °C for denaturation, 1 min at 52 °C for annealing, and 10 min at 72 °C for final extension. The PCR products were purified with the illustra GFX PCR DNA and gel band purification kit (GE Healthcare, Buckinghamshire, UK) and analyzed by Sanger sequencing services (Genewiz Co. Ltd., Saitama, Japan). The DNA sequencing data were analyzed via BLAST searches (performed through the National Center for Biotechnology Information website), and bacterial species were identified based on the highest percentage sequence similarity (> 98%).

### Statistical analyses

The significance of differences in NO_2_^−^-producing activity (per mg wet weight) between dental plaque and tongue coatings or between adults and children were analyzed using Tukey’s test. Similarly, the significance of the differences in the number of NO_2_^−^-producing and non-NO_2_^−^-producing bacteria were analyzed using Tukey’s test. The correlation between the number of NO_2_^−^-producing bacteria and NO_2_^−^-producing activity was examined. P-values of < 0.05 indicated statistical significance. StatFlex Ver. 6 (Artech Co., Ltd, Osaka, Japan) was used for these statistical analyses.
